# Effectiveness of implementing a decentralized delivery of hepatitis C virus treatment with direct-acting antivirals: A systematic review with meta-analysis

**DOI:** 10.1371/journal.pone.0229143

**Published:** 2020-02-21

**Authors:** Rodolfo Castro, Hugo Perazzo, Letícia Artilles Mello Mendonça de Araujo, Isabella Gonçalves Gutierres, Beatriz Grinsztejn, Valdiléa G. Veloso

**Affiliations:** 1 Fundação Oswaldo Cruz, Escola Nacional de Saúde Pública Sergio Arouca, Rio de Janeiro, RJ, Brazil; 2 Universidade Federal do Estado do Rio de Janeiro, Instituto de Saúde Coletiva, Rio de Janeiro, RJ, Brazil; 3 Fundação Oswaldo Cruz, Instituto Nacional de Infectologia Evandro Chagas, Rio de Janeiro, RJ, Brazil; Middle East Liver Diseases (MELD) Center, ISLAMIC REPUBLIC OF IRAN

## Abstract

Direct-acting agents (DAAs) for hepatitis C virus (HCV) treatment are safe and highly effective. Few studies described the sustained virologic response rates of treatment conducted by non-specialists. We performed a systematic review and meta‐analysis to evaluate the effectiveness of decentralized strategies of HCV treatment with DAAs. PubMed, Embase, Scopus and LILACS were searched until March-2019. Studies were screened by two researchers according to the following inclusion criteria: HCV treatment using DAAs on real-life cohort studies or clinical trials conducted by non-specialized health personnel. The primary endpoint was the sustained virologic response rate at week 12 after the end-of-treatment (SVR12), which is binary at the patient level. Data were extracted in duplicate using electronic-forms and quality appraisal was performed with the NIH Quality Assessment Tool. Heterogeneity was assessed by I^2^ statistics. Random-effects meta-analysis models were used for pooling SVR12 rates. Publication bias was assessed using funnel plots. Among the 130 selected studies, nine papers were included for quantitative synthesis. The quality-appraisal was good for two, fair for three and poor for four studies. The pooled relative risk (RR) of SVR12 was not statistically different between decentralized strategy and treatment by specialists [RR = 1.05; 95% confidence interval (95% CI): 0.98–1.1; I^2^ = 45% (95% CI: 0–84%), p = 0.145]. SVR12 rate for decentralized HCV treatment was 81% [SVR12 95% CI: 72–89%; I^2^ = 93% (95% CI: 88–96%)] and 95% [SVR12 95%CI: 92–98%; I^2^ = 77% (95% CI: 52–89%)] with intention to treat analysis and per-protocol analysis, respectively. SVR12 rates using DAAs managed by non-specialized health personnel were satisfactory and similar to those obtained by specialists. This new delivery strategy can improve access to HCV treatment, especially in resource-limited settings. PROSPERO #: CRD42019122609.

## Introduction

Hepatitis C virus (HCV) therapy was revolutionized by use of safe and highly effective direct-acting agents (DAAs) [1, 2]. Treatment with DAAs is associated with reduced risk for mortality and hepatocellular carcinoma and should be considered in all HCV-infected patients [3]. The World Health Organization (WHO) defined strategies to eliminate HCV and to reduce viral hepatitis related deaths by 2030 [4]. However, one of the main barriers to improving HCV care is the lack of an effective linkage-to-care policy for HCV infection involving treatment by non-specialists [5]. In the pegylated interferon (Peg-IFN) era, specialists in gastroenterology or hepatology were the most frequent prescribers of HCV treatment in the United States of America (USA). However, in the last years, HCV treatment managed by infectious disease specialists, internists, general practitioners (GPs) has been increased due to the safety of the new regimens [4, 6]. In order to reach WHO goals in the next decade, non-specialists can play a major role in HCV treatment and long-term follow-up [7].

Several strategies to scale up HCV treatment have been proposed, such as the universal access to highly effective, well-tolerated and affordable regimens. Delivery of treatment using DAAs can involve less trained professionals at lower level health facilities [8]. Implementing new delivery strategies to improve patient access to DAAs in resource-limited settings is crucial to expand testing and treatment to eliminate HCV by 2030 as proposed by the WHO [9]. However, few studies reported relatively low sustained virologic response (SVR) rates (47–66%) of HCV treatment with DAAs conducted by non-specialists in real-world primary care settings [10, 11].

To scale up the decentralization of HCV treatment with DAAs prescribed by non-specialized personnel at primary care settings, evidence synthesis studies need to be conducted to show the effectiveness and safety of these strategies. The aim of this study was to perform a systematic review and meta‐analysis to assess the effectiveness of decentralized strategies of HCV treatment using DAAs.

## Materials and methods

### Registration, search strategy and eligibility criteria

The protocol of this review is at the international prospective register of systematic reviews (PROSPERO #: CRD42019122609) in the following web address: http://www.crd.york.ac.uk/PROSPERO/display_record.php?ID=CRD42019122609

Literature search strategies were conducted in PubMed, Embase, Scopus, LILACS until March-2019 by experienced researchers, with no language or publication period restrictions (S1 Table). References checking, hand searching and contact with authors were strategies used to identify additional studies. After the removal of duplicate studies, titles and abstracts were independently screened by two trained researchers using the Rayyan QRCI web application (https://rayyan.qcri.org/) [12]. Discordances were solved in a panel with the participation of two experienced reviewers. The decision of an experienced hepatologist solved persistent disagreements. The following pre-specified eligibility criteria was adopted: HCV treatment using DAAs on real-life cohort studies or clinical trials at primary care settings, conducted by non-specialized health personnel (GP, family doctor, or any professional without specialization) with description of SVR rates. Cure of HCV was defined by sustained virologic response 12 weeks after the end-of-treatment (SVR12), which is a binary outcome at the patient-level. A detectable HCV viral load 12 weeks after treatment characterized failure of treatment (no SVR12). Studies were included regardless of presenting a comparison group that received the standard specialized care, managed by hepatologists or infectious diseases specialists. Studies were excluded if the primary endpoint, SVR12, was not showed or disaggregated to allow the assessment of the specific effectiveness of the decentralized treatment delivery strategies (S2 Table).

### Data extraction

Study data were extracted in duplicate and managed using electronic data capture tools. Electronic extraction forms were created using REDCap (Research Electronic Data Capture), a secure web-based application designed to support data capture for research studies [13]. SVR12 rates of included studies considering the intention to treat (ITT) and/or the per-protocol analysis were extracted.

### Risk of bias assessment

Quality appraisal was performed using the “Quality Assessment Tool for Observational Cohort and Cross-Sectional Studies Personnel” from the National Institutes of Health (https://www.nhlbi.nih.gov/health-topics/study-quality-assessment-tools). The 14-item checklist on this form was designed to focus on the key concepts for evaluating the internal validity of a study. The quality of studies was rated as good, fair or poor.

### Data analysis

Heterogeneity between the included studies was measured by I^2^ statistics with the following cut-off points were used to classify heterogeneity: 25–50%, 50–75% and >75% considered as mild, moderate and severe, respectively. The “heterogi” [14] program was used to obtain 95% confidence intervals (95% CI) for I^2^ in relative risk meta-analysis and “metaan” [15] for meta-analysis of proportions of SVR12. Random effects models using the method of DerSimonian & Laird, with the estimates of heterogeneity being taken from the Mantel-Haenszel models were used to pool the risk ratio (comparison between decentralized versus specialized strategies and among patients with and without cirrhosis) using “metan” command [16]. Maximum likelihood random effects models (ml) were used for pooling SVR12 rate of decentralized HCV treatment strategies and to estimate heterogeneity using “metaan” [15]. We performed subgroup analyses to explore the heterogeneity and to assess how the pre-identified variables affected the pooled estimates. In addition, fixed effects models were planned to be used only after the identification of an absence of heterogeneity using random-effects models. Results with the same data used in “metaan” for meta-analysis of proportion of SVR12 were additionally presented using random effects models (method of DerSimonian and Laird, with the estimate of heterogeneity being taken from the inverse-variance fixed-effect model) with pooled estimate after Freeman-Tukey Double Arcsine Transformation to stabilize the variances, conducted using “metaprop” [17] Stata program. Funnel plots and Egger’s test assessed publication bias and small-study effects, respectively for analysis of relative risks of SVR12 with two groups’ comparison using “metafunnel” [18]. Additional funnel plots were presented considering publication year using “metabias” [19]. The significance level adopted was 5% and statistical analyses were conducted using the “metan” [16] and “metaprop” [17], “metaan”[15], “metafunnel” [18] and “metabias” [19] packages from Stata-SE (2017; StataCorp LP, College Station, TX, USA).

## Results

### Study characteristics

**Fig 1:**
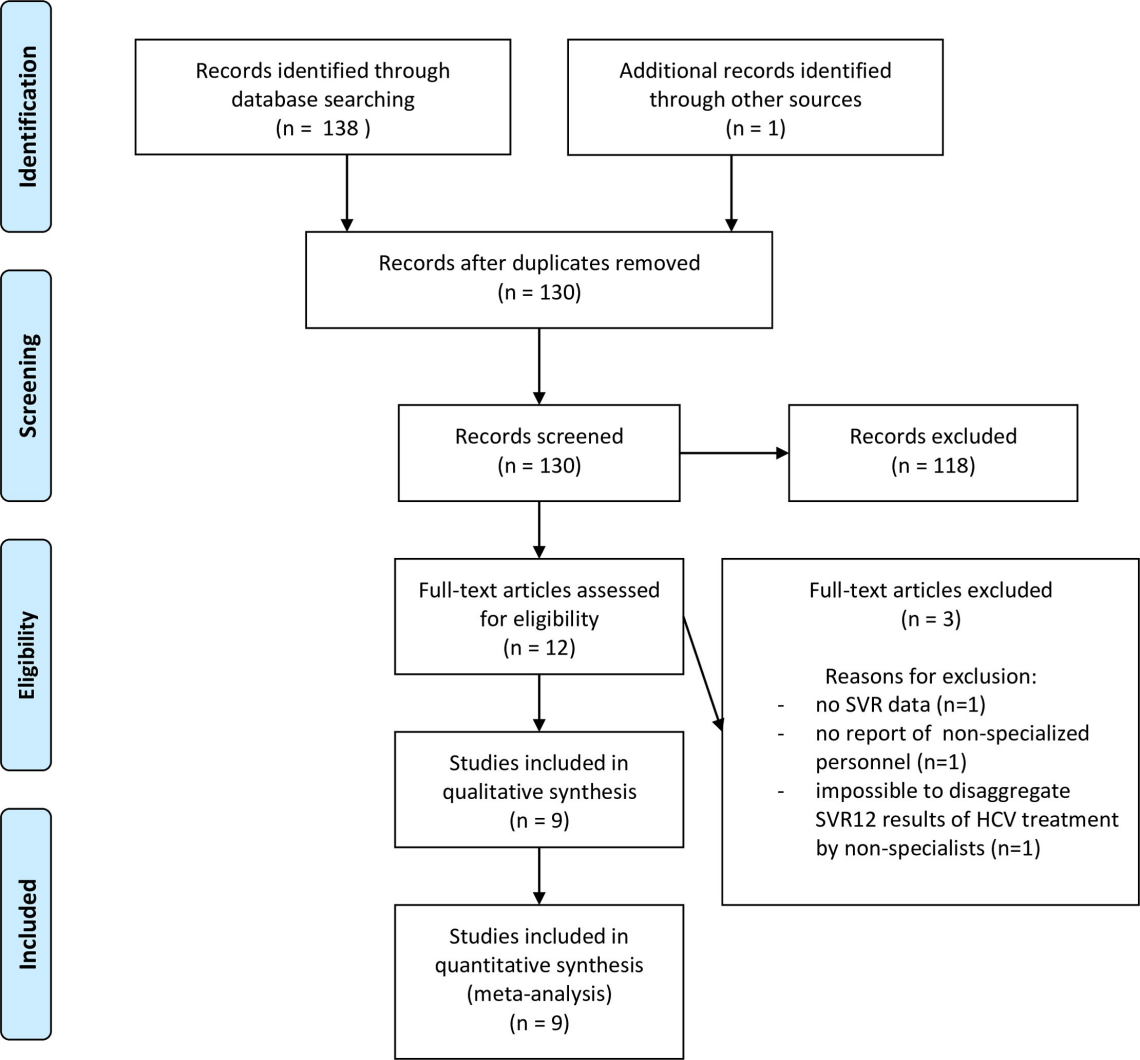
Flow diagram of the study selection process.

The literature search resulted in 130 unique studies (S1 Table). After the screening of titles and abstracts by two blinded investigators, 12 studies [7, 10, 11, 20–28] were eligible for full-text analysis. The percent agreement and Cohen’s kappa between the two independent reviewers were 95% and 0.69, respectively. Nine papers [10, 11, 20, 22–25, 27, 28] fulfilled our eligibility criteria and were finally included after full-text reading. The flow-chart describes details on the selection process (Fig 1).

Overall, 2,099 individuals were treated in the included studies [n = 1,479 by decentralized strategies / n = 620 by specialized delivery of care]. The number of the included patients in the meta-analysis of the proportion of SVR12 with the decentralized strategy was 1,173 for ITT analysis and 917 for per-protocol analysis, considering that some studies showed results of both types of analyses.

Considering the three manuscripts [10, 23, 24] that compared both strategies, HCV treatment with decentralized strategies was delivered for 711 patients, while 620 received specialized care. Among these three studies, only one manuscript had the reporting of SVR12 rates for both ITT and per-protocol analysis [10].

Overall, studies included patients from five countries: USA (n = 3), Australia (n = 3), Pakistan (n = 1), Canada (n = 1), and Rwanda (n = 1). Sofosbuvir (SOF) was the most used DAA, which was present in all included studies regimens. The duration of HCV treatment was for 12 or 24 weeks but was not available for five studies. HCV patients with genotype 1 patients were the most frequently included. Several types of health personnel were used in the decentralization strategies (e.g. mid-level practitioners, social workers, nurses, general practitioners, sexual health physicians, family doctors, and internists) and different types of specialized support (e.g. telephone, electronic messaging, e-mail, education session, remote consultation, pre/post-treatment assessment, and referral to visit by clinical need). Table 1 summarizes the characteristics of included studies.

**Table 1:** Characteristics of the included studies.

Study	Country	Period	Regimen	Duration	Genotypes	Types of Analysis	Non-specialized personnel characteristics	Type of support from specialists (when available)	Specialized personnel characteristics (when the comparison is available)
Jayasekera et al 2015[22]	USA	Dec-2013	SOF /RBV	12 or 24 weeks	GT1	ITT and per-protocol	Part-time licensed vocational nurse (mid-level provider)	Pre and post-treatment assessments, and referral by a clinical need	NA
					GT2				
					GT3				
					GT4				
		Nov-2014	SOF/SIM						
Capileno et al 2017[20]	Pakistan	Feb-2015	SOF /RBV	12 or 24 weeks	GT2	ITT	Mid-level health practitioners	NA	NA
		Dec-2015			GT3				
					GT4				
Kattakuzhy et al 2017[23]	USA	Jan-2015	SOF/LDV	12 weeks	GT1	ITT	Licensed nurse practitioner or physician board-certified in family or internal medicine	NA	Specialist (infectious diseases or gastroenterology or hepatology)
		Nov-2015							
Lasser et al 2017[11]	USA	Mar-2015	NA	NA	NA	ITT and per-protocol	Primary care physicians	Telephone and electronic messaging	NA
		Apr-2016							
Baker et al 2018[27]	Australia	Mar-2016	SOF/DCV	NA	GT1	ITT and per-protocol	General practitioners	Consultation with specialist	NA
		Apr-2016	SOF/LDV		GT3				
Gupta et al 2018[28]	Rwanda	Feb-2017	SOF/LDV	12 weeks	GT1	ITT and per-protocol	Non-specialist clinicians, internists, general practitioner, nurses and social workers	Supervision and mentoring by one internist with specialized HCV training	NA
		Sep-2018			GT4				
Lee et al 2018[24]	Australia	Feb-2016	SOF /RBV	NA	GT1	Per-protocol	General practitioners, sexual health physicians, general physicians, and substance use service	Phone or email support and education sessions	Gastroenterologists
			SOF/DCV		GT2				
					GT3				
			SOF/LDV		GT4				
		Dec-2017			Others				
			SOF/VEL						
Nouch et al 2018[25]	Canada	Oct-2015	SOF /RBV	NA	GT1	ITT and per-protocol	Family doctors	Visit with a specialist when needed	NA
			SOF/LDV ± RBV		GT2				
		Oct-2017			GT3				
			SOF/VEL		GT4				
Wade et al 2018[10]	Australia	July-2015	SOF /RBV	NA	GT1	ITT and per-protocol	General practitioners	Remote specialist consultation	Specialist (infectious diseases or gastroenterology or hepatology)
			SOF/DCV						
			SOF/LDV						
		Jun-2017			GT3				

USA, United States of America; SOF, sofosbuvir; RBV, ribavirin; SIM, simeprevir; LDV, ledipasvir; DCV, daclatasvir; VEL, velpatasvir; NA, not available; GT, genotype; ITT, intention to treat.

### Risk of bias assessment

The quality appraisal assessed by NIH Quality Assessment Tool among the included studies was good for two studies [23, 28], fair for three [22, 24, 25], and poor for four studies [10, 11, 20, 27] (S3 Table). Low participation rate, limited sample and absence of sample size calculation were issues for four studies.

### Risk of SVR12 of decentralized versus specialized strategies

**Fig 2:**
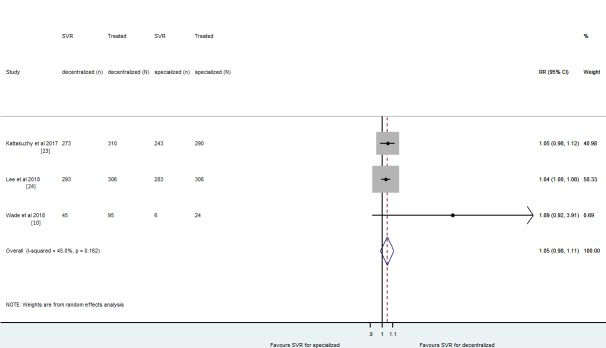
The pooled relative risk of SVR12 for decentralized versus specialized strategies were reported by intention-to-treat (Kattakuzhy et al 2017 and Wade et al 2018) or per-protocol analysis (Lee et al 2018).

The pooled relative risk (RR) of SVR12 was not statistically different between decentralized strategy and treatment by specialists [RR = 1.05 (95% CI: 0.98–1.1); I^2^ = 45% (95% CI: 0–84%); p = 0.145]. This synthesis included the three studies [10, 23, 24] that provide data on the comparison of SVR12 between decentralized and specialized strategies. Overall, 1,331 patients were treated in these studies. The pooled results considered all the types of analysis (ITT and per-protocol). Of these, 85.9% (611/711) had SVR12 with decentralized and 85.8% (532/620) with specialized HCV treatment (Fig 2). The funnel plots, even with presence of some asymmetry did not show significant evidence of publication bias, but the small number of studies limits the interpretation. The Egger's test obtained a p-value of 0.07 and an intercept of 1.67 representing no small-study effects (S1 Fig). A funnel plot with each paper´s publication year of was presented additionally (S2 Fig).

### The effect size of SVR12 rate of decentralized HCV treatment strategies

**Fig 3:**
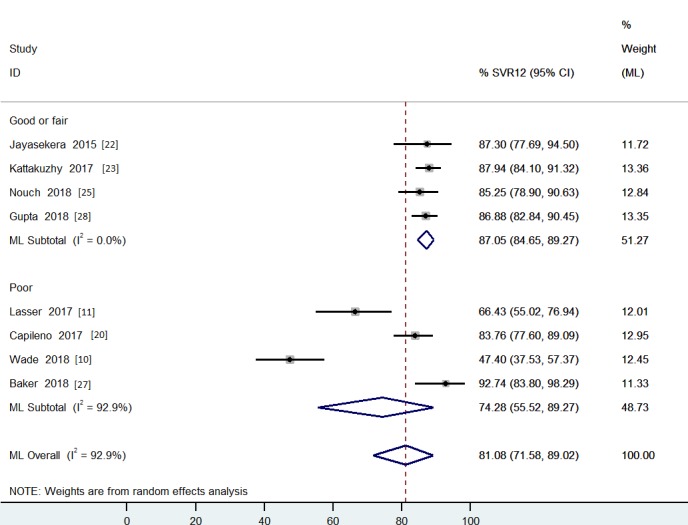
The pooled effect size of SVR12 for decentralized strategy with ITT analysis grouped by studies’ quality appraisal.

**Fig 4:**
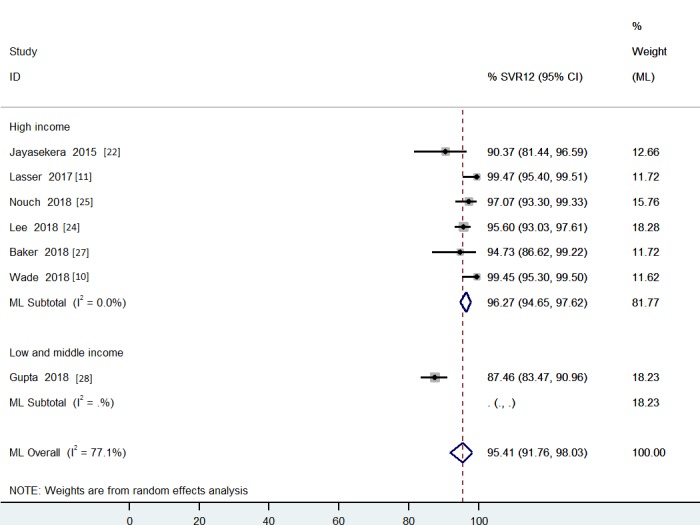
The pooled effect size of SVR12 for decentralized strategy with per-protocol analysis grouped by studies’ country income.

The pooled SVR12 rate for decentralized HCV treatment was 81% [SVR12 95% CI: 72–89%; I^2^ = 93% (95% CI: 88–96%)] and 95% [SVR12 95%CI: 92–98%; I^2^ = 77% (95% CI: 52–89%)] by ITT analysis and by per-protocol analysis, respectively. Heterogeneity was severe among the included studies. The lower individual study point-estimate of SVR12 rate was 47% for results using ITT analysis [10] (Fig 3 and S3 Fig) and the highest were 99% [10, 11] for per-protocol analysis (Fig 4 and S4 Fig). A lower heterogeneity was observed after stratifying studies by risk of bias [I^2^ = 0% (95% CI: 0–85%) when grouping fair/good quality studies] by ITT analysis. The SVR12 rate was 87% (95%CI: 85–89%) (Fig 3). A total number of 806 of patients were included in the “Good or Fair” quality subgroup for sensitivity analysis (S5 Fig).

For per-protocol analysis, the heterogeneity was not resolved by subgroup analysis using the levels of quality (S6 Fig). One study from Rwanda [28] had good quality and a lower SVR12 rate in comparison with the others. The heterogeneity decreased when the studies where grouped by levels of country income. For per-protocol analysis, the final pooled SVR12 rate for decentralized HCV treatment was 96% [SVR12 95% CI: 95–98%; I^2^ = 48% (95% CI: 0–75%)] (Fig 4), including studies with 617 patients in the higher income subgroup (S7 Fig).

In addition, we have found similar results with the same data used for Figs 3 and 4 using random effects models, method of DerSimonian and Laird, with the estimate of heterogeneity being taken from the inverse-variance fixed-effect model with pooled estimates after Freeman-Tukey Double Arcsine Transformation in “metaprop” Stata program (S5 Fig and S7 Fig).

### SVR results of patients with and without cirrhosis treated with decentralization

**Fig 5:**
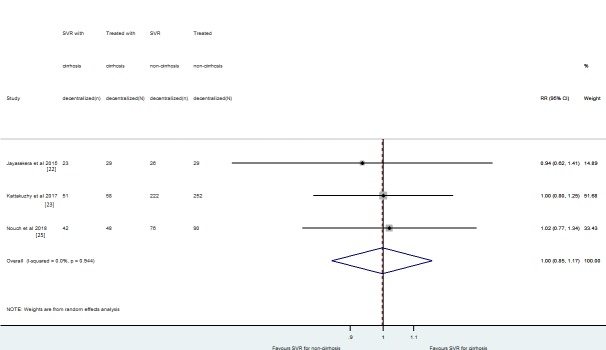
The pooled risk of SVR12 for decentralized strategy, considering patients with cirrhosis versus patients without cirrhosis.

Data extraction on SVR12 rates stratified by absence or presence of cirrhosis was feasible only in three studies using ITT analysis [22, 23, 25]. The SVR12 rates of patients with and without cirrhosis were 85.9% (n = 116/135) and 87.3% (n = 324/371), respectively. The relative risk of SVR12 rate was 1.00 [RR 95% CI: 0.85–1.17; I^2^ = 0% (95% CI: 0–90%); p = 0.961]. This relative risk represents that SVR12 rate was similar for patients with or without cirrhosis among those who were treated by non-specialists (Fig 5). Albeit with limited interpretation by the small number of studies, funnel plot did not show evidence of publication bias and the Egger's test obtained a p-value of 0.455 and an intercept of -0.63 representing no small-study effects (S8 Fig). An additional funnel plot was presented with publication year information (S9 Fig).

## Discussion

The evidence on the effectiveness and safety of DAAs was showed in real-world studies conducted at different settings and subsequent meta-analyses [1, 29–36]. However, to our knowledge, this is the first systematic review and meta-analysis that evaluates the effectiveness of the delivery of HCV treatment with DAAs by non-specialized health personnel at primary care. This meta-analysis highlighted that the HCV treatment using DAAs by non-specialized health personnel was effective and SVR rates were similar to treatment conducted by specialists.

The WHO estimates that up to 71 million people are chronically infected by HCV worldwide [37]. The low screening rates, lack of effective linkage-to-care policies for HCV infection and high drug costs are main barriers for HCV elimination. Many individuals who are unaware that they are HCV positive until the disease progresses into cirrhosis and its complications. HCV cascade can be improved by nation-wide HCV awareness campaign targeting high-risk groups, reduced drugs prices and establishment of multidisciplinary teams to secure linkage to care [38]. Therefore, HCV treatment by non-specialists is a key-strategy to reduce the burden of HCV infection worldwide [39].

Our study reported that SVR12 rates using DAAs by GPs and primary care health personnel are similar to rates obtained by gastroenterologists/hepatologists, reinforcing that this strategy might be implemented in resource-limited setting countries. We acknowledge that patients with cirrhosis should be managed by specialists. Patients with cirrhosis have higher rates of adverse events during treatment [36] and remain at risk of liver-related complications, such as hepatocellular carcinoma after HCV cure [40]. In our systematic review, a small number of patients (n = 134) with cirrhosis were treated by non-specialists. There was a non-significant difference on the SVR rates of patients with versus without cirrhosis treated by non-specialists [RR = 1.0 (95% CI: 0.85–1.17)]. Moreover, the SVR12 was 86.6% for individuals with cirrhosis treated by non-specialists. In our meta-analysis, the presence of cirrhosis has not impacted the primary outcome of HCV treatment. However, patients with cirrhosis should be managed by hepatologist rather than non-specialists due to the higher risk of adverse events during treatment and the need of maintenance of hepatocellular carcinoma screening during a longitudinal follow-up [41]. On the other hand, patients without cirrhosis (METAVIR F≤3) can be managed by GPs or primary care doctors. People with HIV and/or HBV coinfections and other comorbidities (e.g. haemophilia, thalassemia, kidney disfunction) should be referred to specialists [42, 43].

Our meta-analysis has found a considerable difference between ITT and per-protocol results for HCV treatment conducted by non-specialists. Higher lost to follow up (LTFU) is expected at real-world settings, albeit is still possible to find papers reporting similar LFTU between clinical trials and real-world data [44, 45]. Previously, gaps of SVR results for real-world treatment in comparison with clinical trials were due to the LTFU [30]. Moreover, LTFU was related to treatment failure in difficult to treat patients [46]. Future studies should develop and implement new strategies to tackle the LTFU in HCV treatment.

Different decentralization strategies were adopted in the included studies. Only two authors did not mention any type of support offered by specialized personnel [20, 23]. Several categories and combination of health personnel were involved, specialist support or consultancy at distance, training possibilities, and mentoring. Telemedicine support by specialists can be an effective intervention for HCV treatment decentralization to primary care [7].

The main limitations of our study were the high heterogeneity for pooled overall SVR rates and the limited number of studies that compared the decentralized HCV treatment with the standard-of-care. The high heterogeneity could be explained by methodological quality and study design; population and setting characteristics from different countries where the studies were performed, and the variability of specialized support for HCV treatment. As the included studies were investigating the outcomes of new delivery strategies for HCV treatment, with previously unknown effectiveness, the support by specialists could be over implemented due to ethical and/or safety reasons at settings where specialists are available. Apparently, we were able to manage heterogeneity using subgroup analysis considering studies’ quality and income of the country where the studies occurred. Especially in small meta-analyses, it is important to avoid homogeneity assumptions [47]. The report of high heterogeneity rates in our study was an important finding itself. Moreover, the presence of large 95% CI for I^2^, even after achieving a good point estimate through subgroup analysis showed that reporting confidence intervals is very important [48] and approaching heterogeneity can be more challenging than expected. The small number of included studies is a major limitation of our systematic review. Moreover, the assessment of the publication bias that included the presentation of funnel plots is strongly limited. Funnel plots were shown to illustrate this important issue but cannot be used to conclude that publication bias was absent.

The small number of studies included and the heterogeneity results limit the recommendation of treatment decentralization for all settings, but our meta-analysis results contributes to support strategies of decentralized delivery of treatment for key populations, especially in locations where it is not possible to provide specialized care for all people living with HCV. DAAs prescribed and managed by non-specialized health personnel showed good SVR12 rates. The lack of enough specialists for HCV treatment can be tackled with DAA treatment at primary care settings by non-specialized health personnel. Specialists will be necessary to manage patients with specific clinical conditions (e.g. cirrhosis, coinfections, and comorbidities) to reduce the adverse events and improve the treatment efficacy in these cases [40, 42, 43]. Innovative decentralization strategies could be implemented to improve access to HCV treatment, especially in resource-limited settings, contributing to the achievement of HCV elimination targets.

## Supporting information

S1 Table Search activities. (DOCX)

S2 Table Inclusion criteria. (DOCX)

S3 Table Quality assessment using the “Quality Assessment Tool for Observational Cohort and Cross-Sectional Studies Personnel” from the National Institutes of Health (available at: https://www.nhlbi.nih.gov/health-topics/study-quality-assessment-tools). (DOCX)

S1 Fig Funnel plot for the meta-analysis showed in Fig 2, regarding the pooled risk of SVR12 of decentralized versus specialized strategies—Egger's test p-value = 0.07. (TIF)

S2 Fig Funnel plot for the meta-analysis showed in Fig 2, regarding the pooled risk of SVR12 of decentralized versus specialized strategies by publication year. (TIF)

S3 Fig The pooled effect size of SVR12 for all decentralized studies using ITT analysis. (TIF)

S4 Fig The pooled effect size of SVR12 for all decentralized studies using per-protocol analysis. (TIF)

S5 Fig Results with the same data used for Fig 3 using random effects models, method of DerSimonian and Laird, with the estimate of heterogeneity being taken from the inverse-variance fixed-effect model with pooled estimates after Freeman-Tukey Double Arcsine Transformation in “metaprop” Stata program. (TIF)

S6 Fig The pooled effect size of SVR12 for decentralized strategy with per-protocol analysis grouped by studies’ quality appraisal. (TIF)

S7 Fig Results with the same data used for Fig 4 using random effects models, method of DerSimonian and Laird, with the estimate of heterogeneity being taken from the inverse-variance fixed-effect model with pooled estimates after Freeman-Tukey Double Arcsine Transformation in “metaprop” Stata program. (TIF)

S8 Fig Funnel plot for the meta-analysis showed in Fig 5, regarding the pooled risk of SVR12 for decentralized strategy, considering patients with cirrhosis versus patients without cirrhosis—Egger's test p-value = 0.455. (TIF)

S9 Fig Funnel plot for the meta-analysis showed in Fig 5, regarding the pooled risk of SVR12 for decentralized strategy, considering patients with cirrhosis versus patients without cirrhosis by publication year. (TIF)

S1 File PRISMA checklist. (DOC)
