# Supplementary material for: Effectiveness of implementing a decentralized delivery of hepatitis C virus treatment with direct-acting antivirals: A systematic review with meta-analysis
Source: PLoS One. 2020 Feb 21;15(2):e0229143. doi: 10.1371/journal.pone.0229143 (PMC7034833; doi:10.1371/journal.pone.0229143)
Supplement: S1 Table — (DOCX) [file pone.0229143.s001.docx]

**S1 Table.** Search activities.

| **My research question:** | **Effectiveness of Decentralization of HCV treatment with direct-acting agents (DAAs)** | | |
| --- | --- | --- | --- |
| **Databases** | **PubMed, Embase, Scopus, LILACS** | | |
| **List of sources searched:** | **Date of search** | **Search strategy used, including any limits** | **Results** |
| PubMed | 31/Mar/19 | (("hepatitis c"[All Fields] OR HCV[All Fields]) AND (sofosbuvir OR sovaldi OR simeprevir OR olysio OR daclatasvir OR daklinza OR ledipasvir OR harvoni OR elbasvir OR grazoprevir OR zepatier OR velpatasvir OR epclusa OR direct-acting agents) AND (“Primary Health Care"[MeSH Terms] OR "Physicians, Primary Care"[MeSH Terms] OR “General Practitioners"[MeSH Terms] OR “Family Health"[MeSH Terms] OR “General Practice"[MeSH Terms] OR Decentrali*[All Fields]) | 39 |
| Embase | 31/Mar/19 | (('hepatitis'/exp OR hepatitis) AND c OR hcv) AND (sofosbuvir OR sovaldi OR simeprevir OR olysio OR daclatasvir OR daklinza OR ledipasvir OR harvoni OR elbasvir OR grazoprevir OR zepatier OR velpatasvir OR epclusa OR 'direct acting') AND agents AND (((((decentrali* OR primary) AND health AND care OR primary) AND care OR general) AND practitioners OR family) AND health OR general) AND practice | 60 |
| Scopus | 31/Mar/19 | Show results for: ( ( ( TITLE-ABS-KEY ( "hepatitis C" ) ) OR ( TITLE-ABS-KEY ( hcv ) ) ) AND ( ( TITLE-ABS-KEY ( foscavir ) ) OR ( TITLE-ABS-KEY ( vivaldi ) ) OR ( TITLE-ABS-KEY ( simpler ) ) OR ( TITLE-ABS-KEY ( physio ) ) OR ( TITLE-ABS-KEY ( abacavir ) ) OR ( TITLE-ABS-KEY ( dakin ) ) OR ( TITLE-ABS-KEY ( indinavir ) ) OR ( TITLE-ABS-KEY ( harmonic ) ) ) OR ( ( TITLE-ABS-KEY ( alabaster ) ) OR ( TITLE-ABS-KEY ( grapevine ) ) OR ( TITLE-ABS-KEY ( hepatic ) ) OR ( TITLE-ABS-KEY ( nelfinavir ) ) OR ( TITLE-ABS-KEY ( inclusa ) ) OR ( TITLE-ABS-KEY ( "direct-acting agents" ) ) ) ) AND ( ( TITLE-ABS-KEY ( decentrali* ) ) OR ( TITLE-ABS-KEY ( "General Practi*" ) ) OR ( TITLE-ABS-KEY ( "Family Health" ) ) ) | 39 |
| LILACS | 31/Mar/19 | (tw:((tw:(sofosbuvir)) OR (tw:(sovaldi)) OR (tw:(simeprevir)) OR (tw:(olysio)) OR (tw:(daclatasvir)) OR (tw:(daklinza)) OR (tw:(ledipasvir)) OR (tw:(harvoni)) OR (tw:(elbasvir)) OR (tw:(grazoprevir)) OR (tw:(zepatier)) OR (tw:(velpatasvir)) OR (tw:(epclusa)) OR (tw:("direct-acting agents")))) AND (tw:((tw:(Hepatitis C)) OR (tw:(HCV)))) AND (tw:((tw:(Decentrali$)) OR (tw:(General Practi$)) OR (tw:(Primary Care)) OR (tw:(Family Health)))) | 0 |
| Total of references  [Identification] | 31/Mar/19 |  | 138 |
| References without duplicates  [Screening] | 31/Mar/19 |  | 129 |
| Other source reference |  |  | 1 |
| Full texts articles eligible  [Eligibility] |  |  | 12 |
| Studies included  [Inclusion] |  |  | 9 |
