# Supplementary material for: Effectiveness of implementing a decentralized delivery of hepatitis C virus treatment with direct-acting antivirals: A systematic review with meta-analysis
Source: PLoS One. 2020 Feb 21;15(2):e0229143. doi: 10.1371/journal.pone.0229143 (PMC7034833; doi:10.1371/journal.pone.0229143)
Supplement: S2 Table — (DOCX) [file pone.0229143.s002.docx]

**S2 Table.** Inclusion criteria.

| **CRITERIA** | | **NOTES** | |
| --- | --- | --- | --- |
| **Population:**  People living with chronic hepatitis C | | Please include studies of HCV patients with coinfections as HIV, HBV, among others. Just exclude if the patients are not positive to HCV | |
| **Interventions:**  Decentralized HCV treatment using DAAs on real-life cohort studies or clinical trials at settings of primary health care and conducted by non-specialists health professionals (General Practitioner, Family Doctor, or any kind of professional **without** specializations to treat liver and/or infectious diseases) | | Please see table for details of the eligible combinations and licensed doses | |
| **Comparison:**  HCV treatment by specialists (Infectious diseases specialist, Gastroenterologist, Hepatologist, and doctors **with** specializations to treat liver and/or infectious diseases) | | **Non-eligible studies:** studies of HCV prevalence or screening; clinical trials or cohort studies that evaluated the effectiveness of DAAs only conducted by specialized professionals | |
| **Outcomes:**  Sustained virological response at week 12 after end-of-treatment (SVR12) | | Eradication of HCV virus [sustained virologic response] in intention-to-treat (ITT) and per-protocol analysis | |
| **Study Designs:**  Randomized or open-label clinical trials and real-life cohort studies | | **Eligible studies:** randomized or open-label clinical trials comparing the effectiveness of DAAs or cohort studies reporting the effectiveness of DAAs for HCV eradication | |
| **Products** | **Presentation** | | **Brand names** |
| Sofosbuvir | Tablets containing 400 mg | | Sovaldi |
| Simeprevir | Capsules containing 150 mg | | Olysio |
| Daclatasvir | Tablets containing 30 or 60 mg | | Daklinza |
| Sofosbuvir/ledispavir | Tablets containing 400 mg of sofosbuvir and 90 mg of ledispavir | | Harvoni |
| Sofosbuvir/Velpatasvir | Tablets containing 400 mg of sofosbuvir and 100 mg of velpastavir | | Epclusa |
| Grazoprevir /Elbasvir | Tablets containing 100 mg of grazoprevir and 50 mg of elbasvir | | Zapatier |
| **Regimens** | | | **Duration** |
| Sofosbuvir/Daclatasvir +/- ribavirin | | | 12 or 24 weeks |
| Sofosbuvir/Simeprevir +/- ribavirin | | | 12 or 24 weeks |
| Sofosbuvir/ledispavir +/- ribavirin | | | 8 or 12 weeks |
| Sofosbuvir/Velpatasvir +/- ribavirin | | | 12 weeks |
| Grazoprevir /Elbasvir +/- ribavirin | | | 12 weeks |
