# Supplementary material for: Effectiveness of implementing a decentralized delivery of hepatitis C virus treatment with direct-acting antivirals: A systematic review with meta-analysis
Source: PLoS One. 2020 Feb 21;15(2):e0229143. doi: 10.1371/journal.pone.0229143 (PMC7034833; doi:10.1371/journal.pone.0229143)
Supplement: S3 Table — (DOCX) [file pone.0229143.s003.docx]

**S3 Table.** Quality assessment using the “Quality Assessment Tool for Observational Cohort and Cross-Sectional Studies Personnel” from the National Institutes of Health (available at: https://www.nhlbi.nih.gov/health-topics/study-quality-assessment-tools).

| **Study** | **Q1** | **Q2** | **Q3** | **Q4** | **Q5** | **Q6** | **Q7** | **Q8** | **Q9** | **Q10** | **Q11** | **Q12** | **Q13** | **Q14** | **Quality** |
| --- | --- | --- | --- | --- | --- | --- | --- | --- | --- | --- | --- | --- | --- | --- | --- |
| Jayasekera et al 2015 [22] | Yes | Yes | Yes | Yes | No | Yes | Yes | Yes | Yes | Yes | Yes | NR | Yes | NR | Fair |
| Capileno et al 2017 [20] | Yes | No | No | Yes | No | Yes | Yes | No | No | NR | Yes | NR | Yes | NR | Poor |
| Kattakuzhy et al 2017 [23] | Yes | Yes | Yes | Yes | Yes | Yes | Yes | Yes | Yes | Yes | Yes | NR | Yes | NR | Good |
| Lasser et al 2017 [11] | Yes | Yes | NR | Yes | No | Yes | Yes | No | No | Yes | Yes | NR | No | No | Poor |
| Baker et al 2018 [27] | NR | Yes | NR | Yes | No | Yes | Yes | No | No | No | Yes | No | Yes | NR | Poor |
| Gupta et al 2018 [28] | Yes | Yes | Yes | Yes | Yes | Yes | Yes | Yes | Yes | Yes | NR | NR | Yes | Yes | Good |
| Lee et al 2018 [24] | Yes | No | Yes | Yes | No | Yes | Yes | No | No | No | Yes | NR | Yes | NR | Fair |
| Nouch et al 2018 [25] | Yes | Yes | Yes | Yes | No | Yes | Yes | Yes | Yes | Yes | No | NR | Yes | NR | Fair |
| Wade et al 2018 [10] | Yes | No | Yes | Yes | NR | NR | Yes | No | No | No | No | NR | No | No | Poor |

NR, Not reported
