# Supplementary figures and images for: Effectiveness of implementing a decentralized delivery of hepatitis C virus treatment with direct-acting antivirals: A systematic review with meta-analysis
Source: PLoS One. 2020 Feb 21;15(2):e0229143. doi: 10.1371/journal.pone.0229143 (PMC7034833; doi:10.1371/journal.pone.0229143)

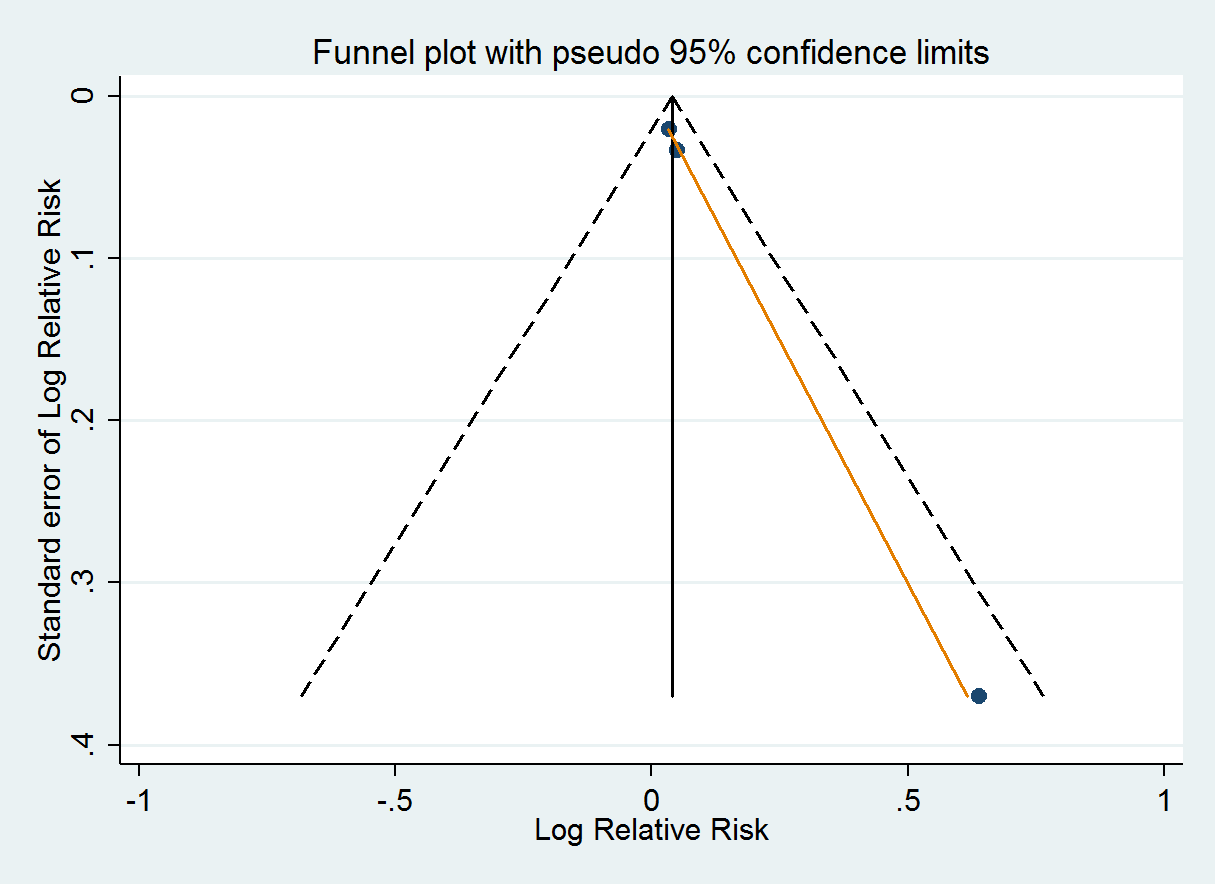

Supplement: S1 Fig — (TIF) [file pone.0229143.s004.tif]

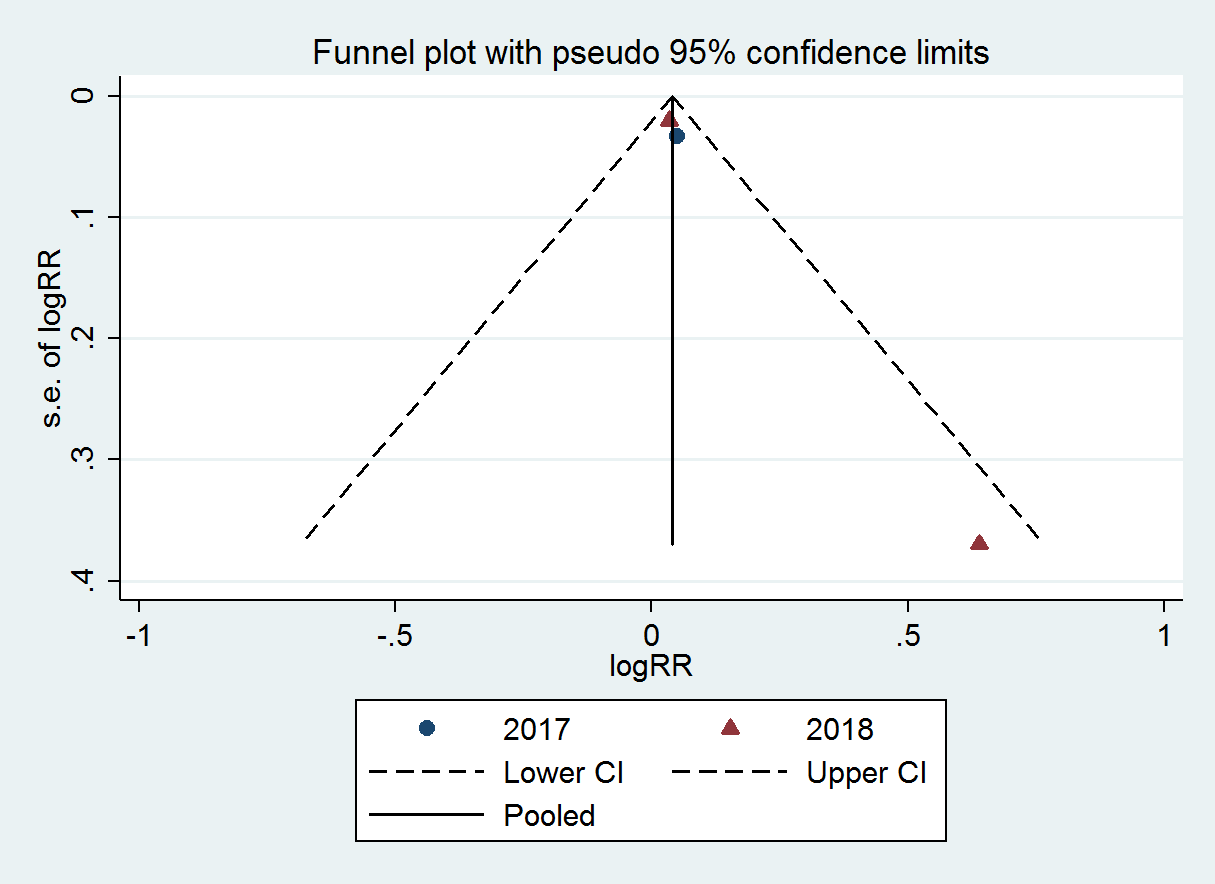

Supplement: S2 Fig — (TIF) [file pone.0229143.s005.tif]

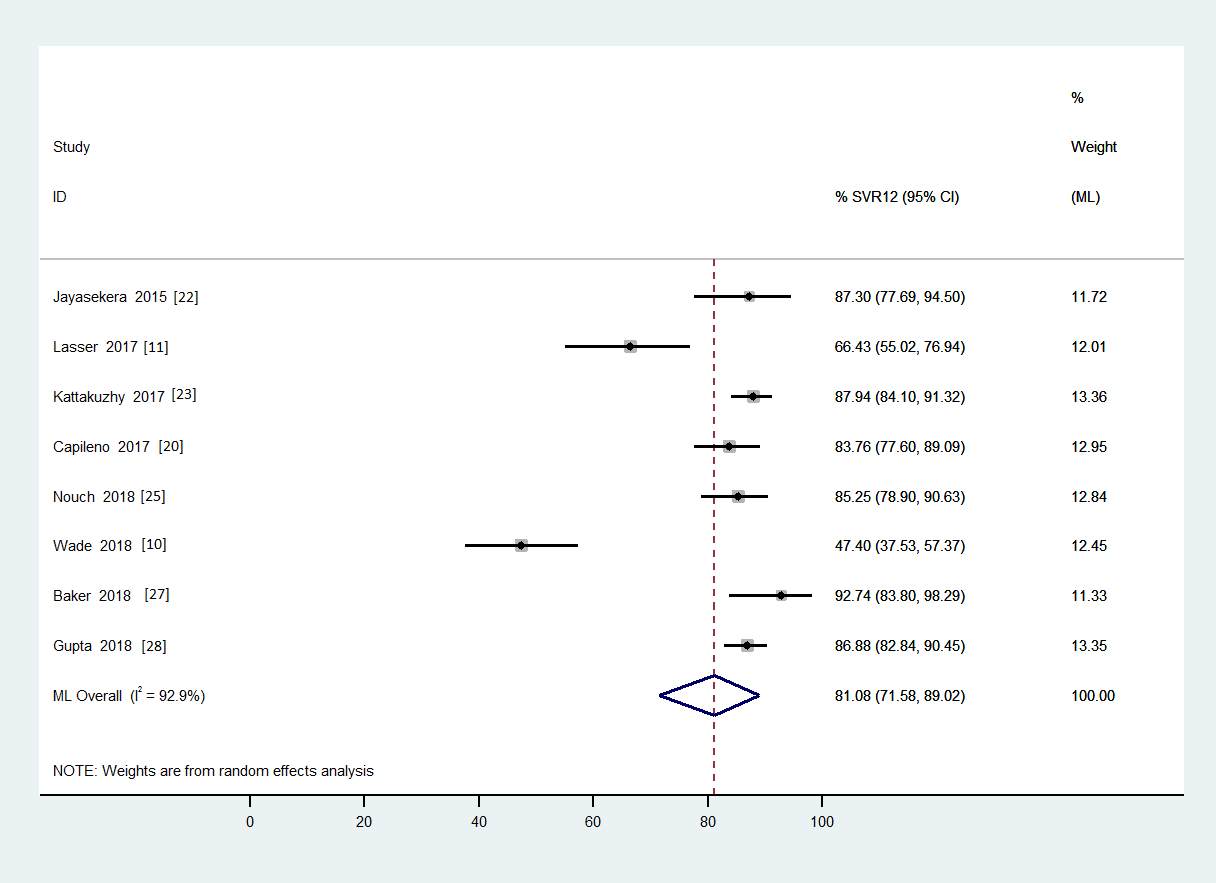

Supplement: S3 Fig — (TIF) [file pone.0229143.s006.tif]

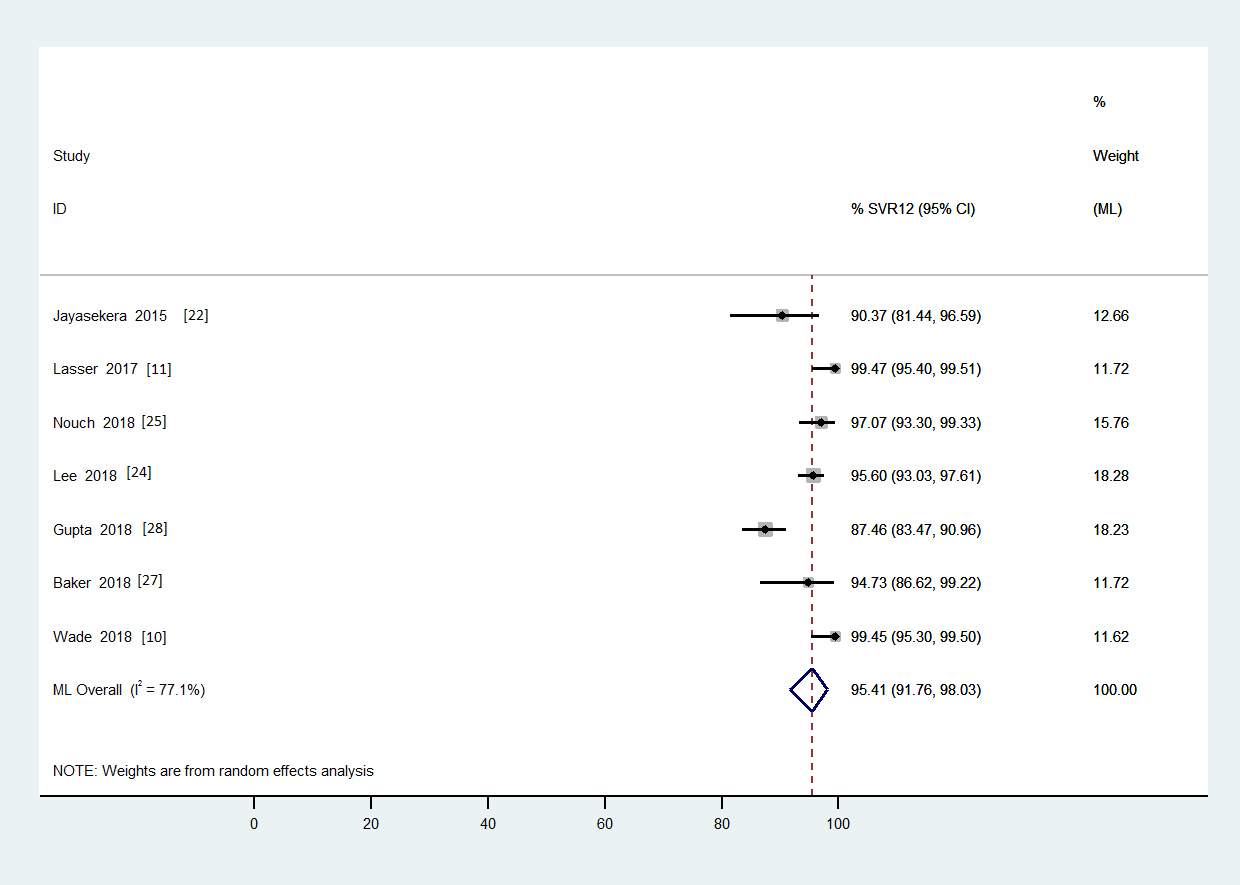

Supplement: S4 Fig — (TIF) [file pone.0229143.s007.tif]

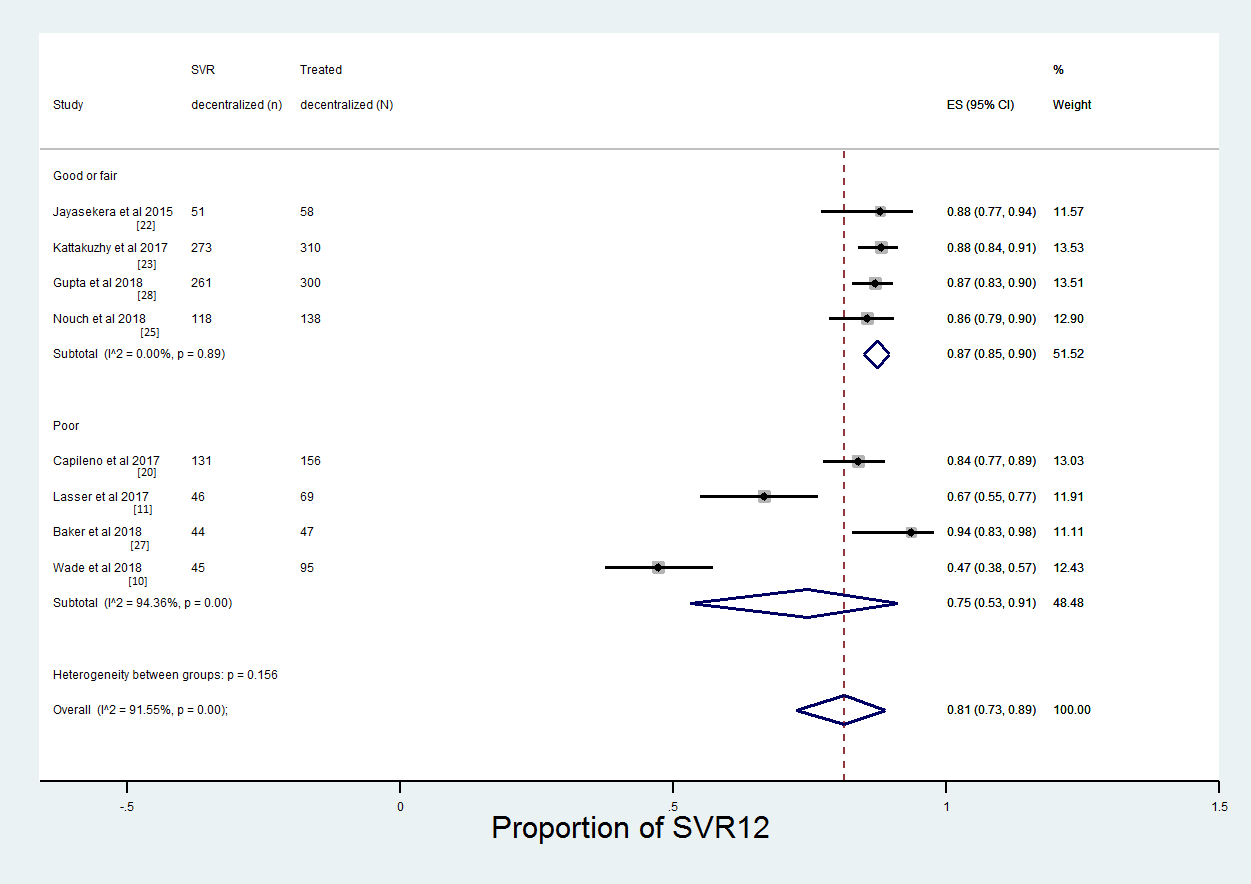

Supplement: S5 Fig — (TIF) [file pone.0229143.s008.tif]

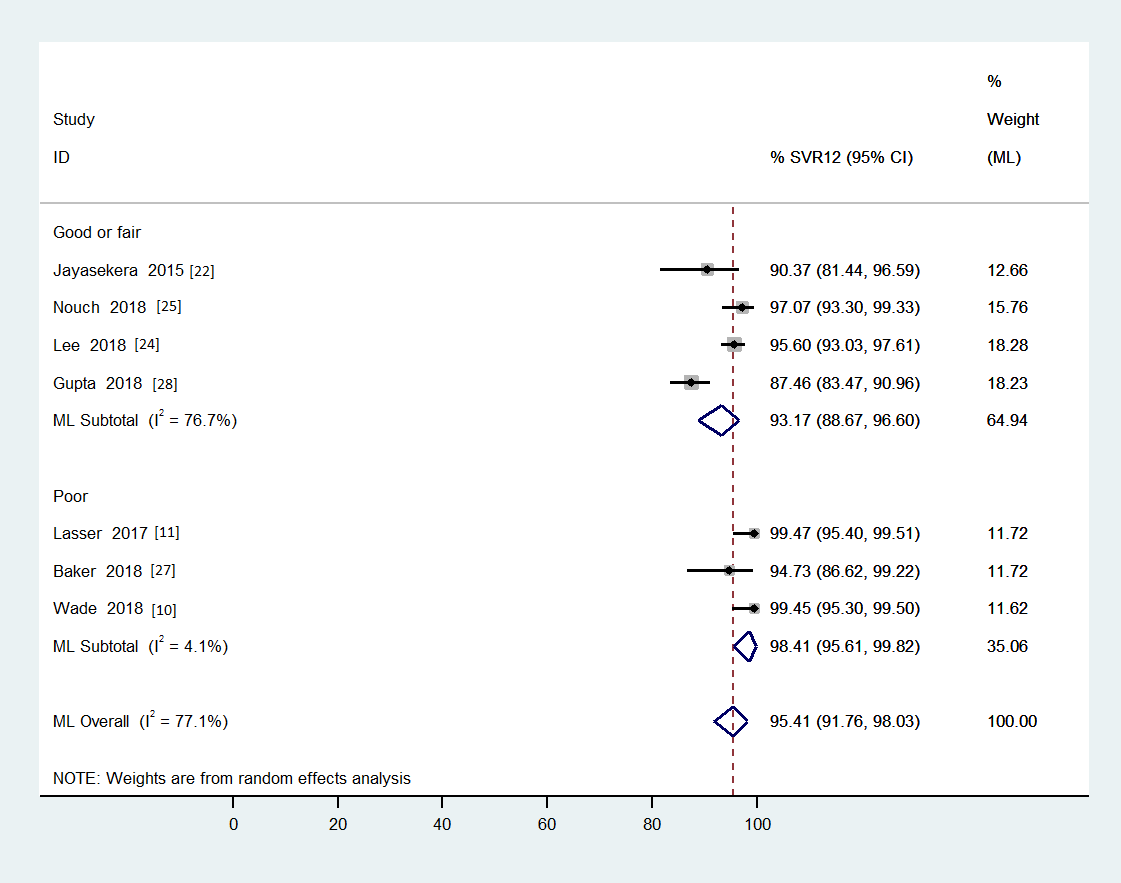

Supplement: S6 Fig — (TIF) [file pone.0229143.s009.tif]

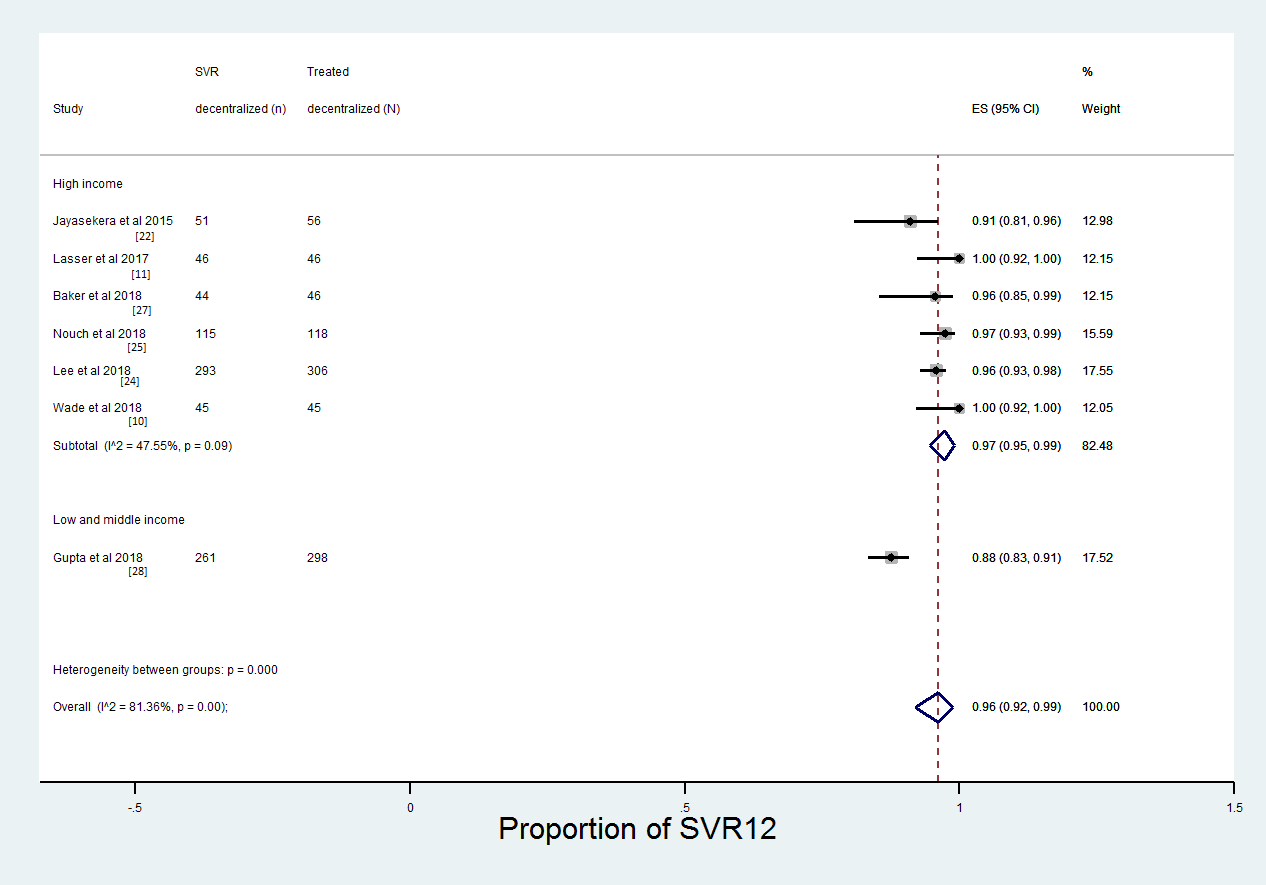

Supplement: S7 Fig — (TIF) [file pone.0229143.s010.tif]

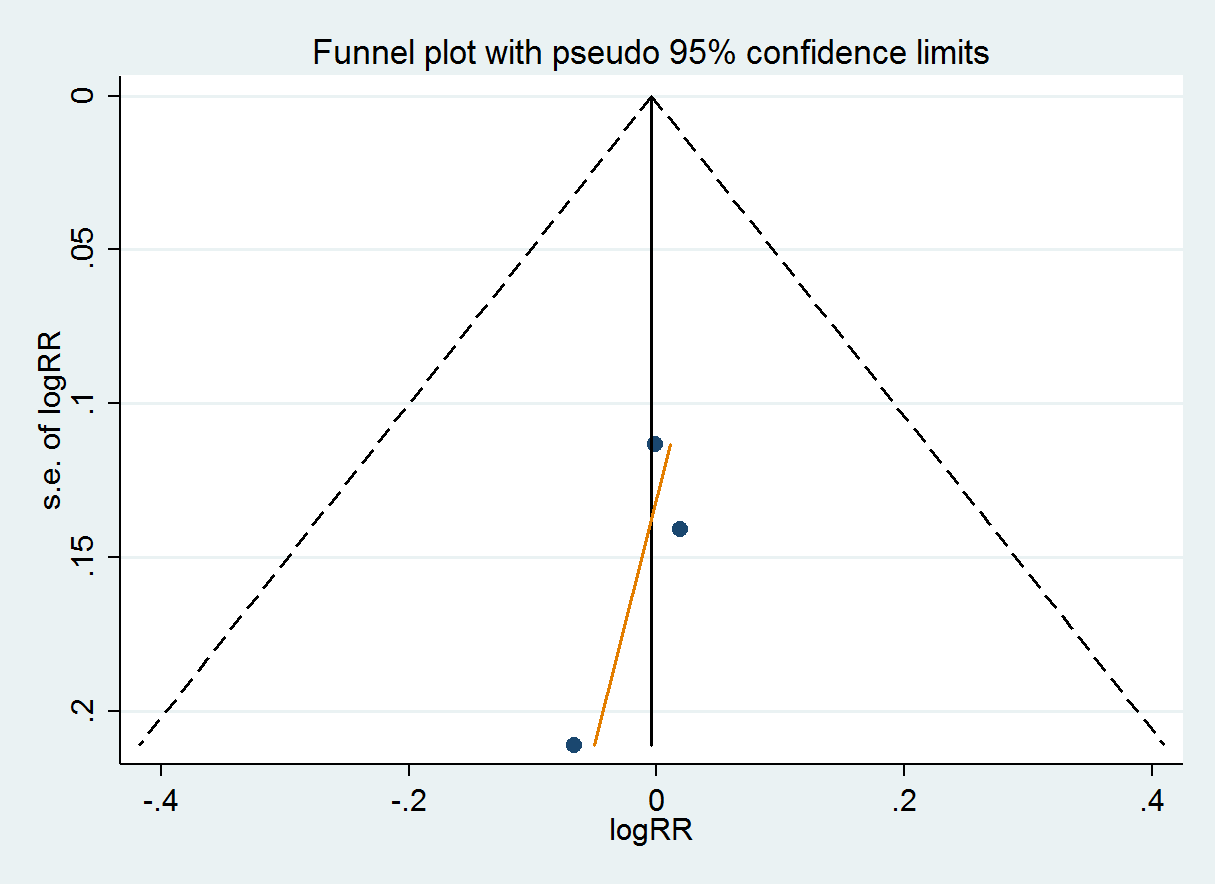

Supplement: S8 Fig — (TIF) [file pone.0229143.s011.tif]

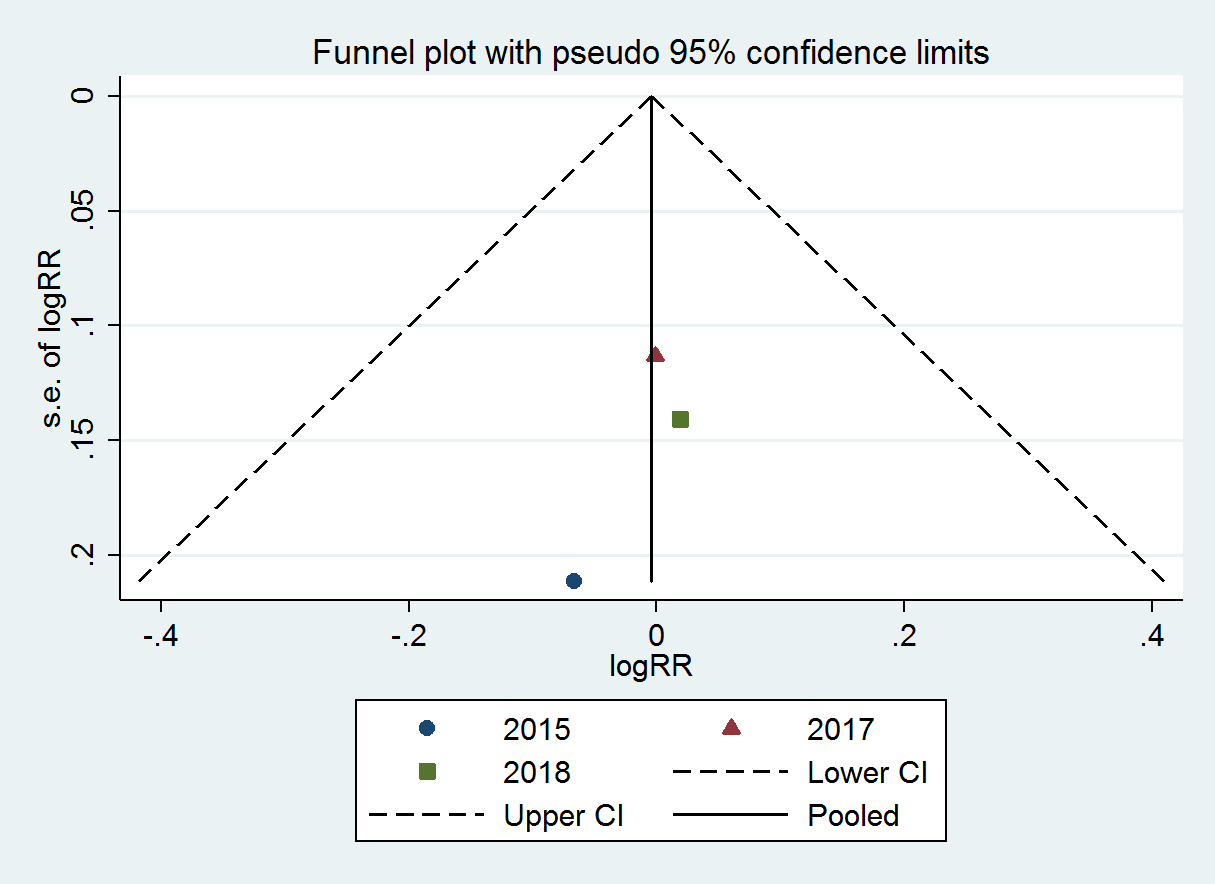

Supplement: S9 Fig — (TIF) [file pone.0229143.s012.tif]
